# Insertion site preference of Mu, Tn5, and Tn7 transposons

**DOI:** 10.1186/1759-8753-3-3

**Published:** 2012-02-07

**Authors:** Brian Green, Christiane Bouchier, Cécile Fairhead, Nancy L Craig, Brendan P Cormack

**Affiliations:** 1 Department of Molecular Biology and Genetics, Johns Hopkins University School of Medicine, Hunterian 617, 725 North Wolfe Street, Baltimore, MD 21205-2185, USA; 2 Institut Pasteur, Plate-forme Génomique, Département Génomes et Génétique, Rue du Dr. Roux, F-75015 Paris, France; 3 Institut de Génétique et Microbiologie, Université Paris Sud 11, CNRS UMR8621, 15 rue Georges Clémenceau, Orsay F-91405, France; 4 Department of Molecular Biology and Genetics, The Howard Hughes Medical Institu725 North Wolfe Street Johns Hopkins University School of Medicine, Baltimore, MD 21205-2185, USA

**Keywords:** Tn7, Mu, Tn5, Mutagenesis, Insertion site, DNA transposon, Mobile element

## Abstract

**Background:**

Transposons, segments of DNA that can mobilize to other locations in a genome, are often used for insertion mutagenesis or to generate priming sites for sequencing of large DNA molecules. For both of these uses, a transposon with minimal insertion bias is desired to allow complete coverage with minimal oversampling.

**Findings:**

Three transposons, Mu, Tn5, and Tn7, were used to generate insertions in the same set of fosmids containing *Candida glabrata *genomic DNA. Tn7 demonstrates markedly less insertion bias than either Mu or Tn5, with both Mu and Tn5 biased toward sequences containing guanosine (G) and cytidine (C). This preference of Mu and Tn5 yields less uniform spacing of insertions than for Tn7, in the adenosine (A) and thymidine (T) rich genome of *C. glabrata *(39% GC).

**Conclusions:**

In light of its more uniform distribution of insertions, Tn7 should be considered for applications in which insertion bias is deleterious.

## Background

Transposons, mobile DNA elements that can integrate into target DNA molecules, are useful for insertional mutagenesis, gene tagging, gene transfer, and sequencing applications. A major class of transposable elements used for genome engineering is DNA 'cut and paste' transposons. The transposases for DNA transposons cut the transposon away from the donor DNA by a variety of mechanisms and the excised transposon integrates into a new target site by joining of its 3'OH termini to staggered positions on the top and bottom DNA strands of the target. This staggered joining results in a target site duplication of a defined number of base pairs, which can be used to map precisely the site of integration for the transposon [1].

In most of the applications of transposons to molecular biology, it is important that the transposon insert into target DNA with little to no sequence bias. Limited sequence bias will lead to more complete coverage of a region for a given number of insertion events. However, most transposons have been shown to exhibit some preference for certain sequences or sequence features [1]. Clearly, insertion site bias may be a confounding factor for large scale transposon mutagenesis projects.

A number of manuscripts reporting insertion motifs for various transposons have been published, but the target DNA, transposition protocol and environment (*in vitro *versus *in vivo*) vary widely, making direct comparisons difficult. For example, individual genes [2], *Escherichia coli *genomic DNA [3], and *Saccharomyces cerevisiae *genomic DNA [4] have been used. In this publication, three transposon systems were evaluated using the same target DNA *in vitro*: Mu, Tn5, and a modified Tn7 [5]. Previous work had identified a CPy(G/C)PuG or similar motif for Mu [6-8], a GPyPyPy(A/T)PuPuPuC motif for Tn5 [9,10] and negligible bias for the modified Tn7 [11]. Since previous publications all used different target DNA, and because our DNA of interest (*C.glabrata *genomic DNA) has a moderately high A/T content (61%, [12]), specificity and distribution of insertion sites for all three transposons was assessed on the same target DNAs.

## Methods

BACs containing *C. glabrata *genomic DNA were prepared as follows. First, the vector plasmid pBAC-NAT was constructed in two steps. pCR2.1-NAT was constructed by amplifying the NAT cassette using primers ON-5'NAT (CCGCTGCTAGGCGCGCCGTGGAAGTTCCTATACTTTCTAGAGAATAGGAACTTCGATCCCCCCCATAAAGCACGTGATAGCTTC) and ON-3'NAT (GCAGGGATGCGGCCGCTGACGAAGTTCCTATTCTCTAGAAAGTATAGGAACTTCAGCTTGATATCGAATTCCGCAAATTAAAGCC) from pCaNAT1 (a gift of Julia Koehler) and cloning that into pCR2.1 using the TA/TOPO kit (Life Technologies, Carlsbad, CA, USA 92008) The NAT cassette was amplified using primers ON3601 (AGTCGCGGCCGCGTTTAAACGGCGCCCCGCTGCTAGGCGCGCCGTG) and ON3602 (AGTCGGCCCGGGCGGCCACGCGTTGACCCGCGGGCAGGGATACGGCCGCTGAC), cloned into pCR2.1, sequence verified, and a NotI/SfiI fragment of that was inserted into pBAC [13] cut with NotI/SfiI to yield pBAC-NAT (pB1895).

Next, genomic DNA was inserted into pBAC-NAT. The four plasmids into which transposons were mobilized contain genomic DNA from *C. glabrata *from the indicated ORF to the telomere. The genomic DNA began at *CAGL0A00187g *from the strain BG2 [14] for pB1907 (24,252 bp and 34% GC insert), from *CAGL0C00297g *and strain BG2 for pB1908 (31,757 bp and 34% GC insert), from *CAGL0C05599g *and strain BG2 for pB1909 (25,125 bp and 34% GC insert), and from *CAGL0C05599g *and strain CBS138 [12] for pB1910 (19,423 bp and 31% GC insert). Although pB1909 and pB1910 contain the region from the same gene to the telomere from different strains, they are only homologous for the centromeric (rightmost in figures) approximately 8 kb, after which they diverge completely (data not shown).

Mu transposition reactions were carried out per the manufacturer's recommendations using the Finnzyme Template Generation System Kit (Thermo Fisher Scientific, Waltham, MA, USA 02454), with pB1909 and pB1910 as target DNA sequences. Tn5 transposition reactions were carried out per the manufacturer's recommendations using the Ez-Tn5 kit (Epicentre, Madison, WI, USA 53713) with pB1907, pB1908, pB1909, and pB1910 as targeting sequences. Tn7 reactions were carried out as published, [13] with pB1907, pB1908, pB1909, and pB1910 as targeting sequences.

All sequencing was done using the ABI BigDye Terminator Kit v1.1 (Life Technologies, Carlsbad, CA, USA 92008). The primers used for sequencing the Mu transposon containing clones were SeqE (CGACACACTCCAATCTTTCC) and SeqW (GGTGGCTGGAGTTAGACATC). The primers used for sequencing the Tn5 transposon containing clones were KAN-2 FP-1 (ACCTACAACAAAGCTCTCATCAACC) and KAN-2 RP-1 (GCAATGTAACATCAGAGATTTTGAG). The primers used for sequencing the Tn7 transposon containing clones were ON661 (ATAATCCTTAAAAACTCCATTTCCACCCCTCCCAG) and ON662 (GACTTTATTGTCATAGTTTAGATCTATTTTGTTCAG).

BLogo sequence logos were generated using the web form at http://www.bioinformatics.org/blogo/cgi-bin/Blogo/Blogoform.pl[15] as type 2 logos with coloring for symbols with *P *< 0.001 (Fisher's exact test) and base representation calculated from the fosmid sequences into which the various transposons were integrated. The background frequencies of A, C, G, and T used for the BLogo sequence logos are given in the figure legends.

## Results

Fosmids containing subtelomeric and telomeric genomic DNA from *C. glabrata *were used as targets for transposon insertion *in vitro *for the transposons Mu, Tn5, and Tn7. Following transformation to select insertions, the resulting clones were individually selected and sequenced from both ends of the transposon. The two reads for each clone were merged to yield the sequence of the ten nucleotides upstream of the transposon mediated duplication, the duplication, and ten nucleotides downstream of the duplication. Table 1 shows the number of these insertion events that could be mapped to locations within the target fosmid.

**Table 1:** Number of insertions mapped

Fosmid	Mu	Tn5	Tn7
pB1907	n/a	97	121

pB1908	n/a	58	113

pB1909	139	73	139

pB1910	113	48	106

Total	252	276	479

**Figure 1:**
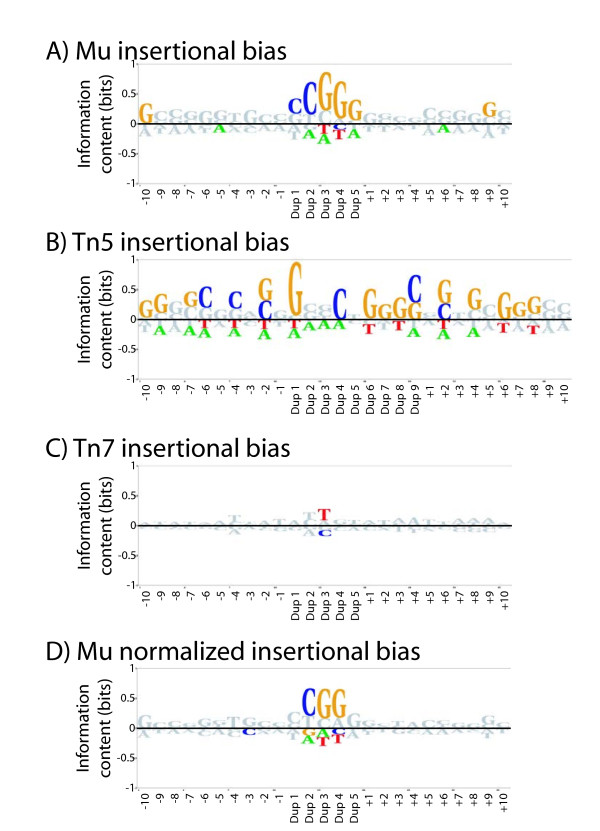
**Insertion biases of Mu, Tn5, and Tn7**. BLogo sequence logos are shown for all insertion sites for Mu **(A)**, Tn5 **(B)**, and Tn7 **(C) **transposons into *Candida glabrata *subtelomeric DNA. Ten bases on either side of the transposon mediated duplication of five (Mu and Tn7) or nine (Tn5) bases were used to generate the sequence logo, and bases significant at *P *< 0.001 are shown in color. Mu insertion events were used to create a BLogo sequence logo with background base frequencies obtained from selecting all 25 mers within the target fosmids containing a central CGG **(D)**. The background frequencies of A, C, G, and T used for the BLogo sequence logos are respectively: 0.32, 0.19, 0.18, 0.31 (A,B,C); 0.275, 0.217, 0.224, 0.284 (D).

All insertion events for a given transposon were used to generate BLogo sequence logo plots of position specific sequence bias, with positions colored if significant at *P *< 0.001 (Figure 1). BLogo sequence plots are a position specific log based measure of the overrepresentation (above the line) or underrepresentation (below the line) of each base at each position around the insertion sites. The plot for Mu insertions (Figure 1A) shows a strong bias for a CGG motif central to the 5 bp duplicated region, which has been previously reported [8]. The Tn5 insertions are also biased (Figure 1B), with a strong preference for G at the first bp of the duplication and a general bias for G and C across the analyzed region. This motif is similar, but not identical, to that previously reported for Tn5 mutagenesis [9]. In contrast, the Tn7 has only a very weak bias, toward a T in the middle position of the duplication site (Figure 1C).

The G/C content of the fosmids mutagenized is not uniform across their length (see below), so some of the apparent bias in the flanking base pairs might be simply due to a sampling bias due to a strongly biased central core. In fact, if the base percentages used in BLogo are calculated from all 25mers in the fosmids containing a central CGG core, no other positions show significant bias for Mu transposition (Figure 1D). The results from a similar analysis for Tn5 were ambiguous, but since many of the Tn5 insertions were in the *C. glabrata *telomeric repeats (which are very G/C rich), this could explain the observed flanking bias.

**Figure 2:**
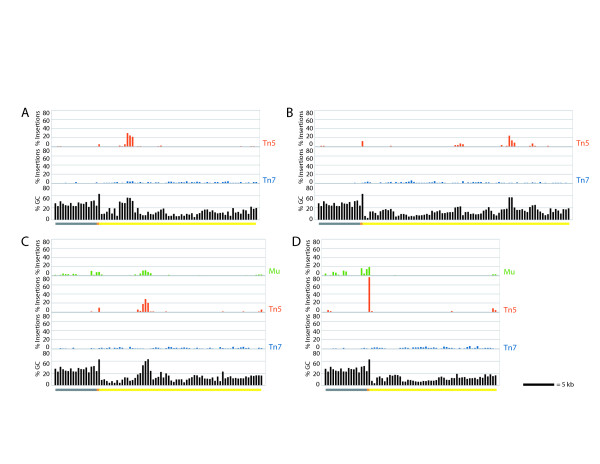
**Insertion events are not uniformly distributed for Mu and Tn5**. Histograms of the percent of insertions contained in each 400 base pair window along the fosmids are shown (green, red, and blue bar graphs) for pB1907 **(A)**, pB1908 **(B)**, pB1909 **(C)**, and pB1910 **(D)**. For each fosmid, the percent G or C in the same 400 base pair windows is shown in black. Grey bars indicate the position of the fosmid backbone, orange bars the telomeric repeats, and yellow bars the subtelomeric DNA along the graphs.

Depending on the nucleotide composition of the target fragment, insertion site bias would be expected to result in non-random spacing of insertions in targets with variable G/C content. Histograms showing the percent of insertions in each 400 bp window spanning the fosmid demonstrate that Mu and Tn5 had strongly clustered insertions (Figure 2). For Tn5, 10% of the 400 bp windows accounted for 92.4% of the insertions; for Mu, it was 72.6%. In contrast, the spacing of Tn7 insertions is much more uniform (Figure 2), with the top 10% of 400 bp windows containing 32.8% of the insertions. The sequence motifs for both Mu and Tn5 are G/C rich; analysis of the percent G/C for each 400 bp window shows that the strongest peaks in the frequency of Mu and Tn5 insertions occur in regions of relatively elevated G/C content (Figure 2).

**Figure 3:**
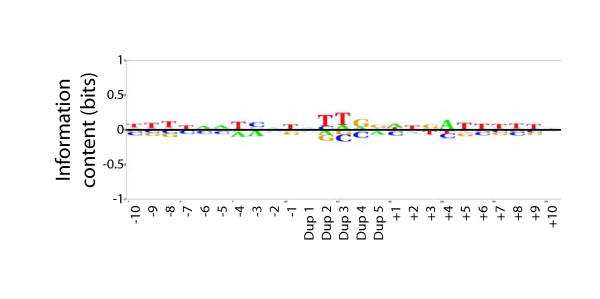
**Tn7 does exhibit a very modest bias toward A/T rich sequences**. A BLogo sequence logo was generated as in Figure 1, using 6,700 Tn7 insertion events from 49 fosmids. The background frequencies of A, C, G, and T used for the BLogo sequence logos are 0.317, 0.196, 0.186, 0.301, respectively.

As part of a larger sequencing effort, and due to the minimal sequence bias discussed above, Tn7 was then used to mutagenize 49 fosmids containing *C. glabrata *subtelomeric genomic DNA. A total of 6,700 insertion events were used to generate a BLogo sequence motif (Figure 3). In contrast to the smaller set of Tn7 insertion events discussed above, and due to the larger number of sequences used, the subtle biases exhibited by Tn7 were significant at *P *< 0.001. Although the relative bias is very weak, Tn7 does exhibit a bias toward a central TTG core. There is also a slight palindromic bias in the flanking regions; positions plus 1 to plus 6 are overrepresented for ATGATT and positions minus 6 to minus 1 for AATCAT. Overall, the slight bias seen for Tn7 is for A/T rich sequences and is markedly less pronounced than the G/C biases of Mu and Tn5.

## Conclusions

Uniform distribution of transposon insertion is important for their use in many molecular biological applications. In the analysis here, Tn7 demonstrates a far more random insertion profile than either Mu or Tn5. For Tn5 and Mu, the consensus sequences derived are consistent with extensive published analyses [5-10]. Transposon insertion site preference is complex. For example, previous studies of Mu insertion have shown that particular dinucleotides, or base steps, contribute to site preference; predicted structural features of DNA also may play a role [6-8]. However, even these comprehensive analyses of insertion site preference derived consensus sites that are both G/C rich and consistent with those we derived here. We suggest that the preference for G/C rich sequences exhibited by Mu and Tn5 is of particular importance for investigators working with A/T rich genomes such as *S. cerevisiae *(approximately 62% A/T) and many other fungi, *Caenorhabditis elegans *(approximately 65% A/T), and human (approximately 60% A/T) [16]. Tn7 has less target bias, with a weak preference for A/T rich sequences. For the large genomic inserts analyzed here, this resulted in a broad distribution of insertion sites, with a bias away from the plasmid backbone (which is more G/C rich relative to the cloned genomic DNA). The minimal sequence bias exhibited by Tn7 suggests that Tn7 should be considered for generating random insertions in cloned A/T-rich genomes.

## Abbreviations

bp: base pair.

## Competing interests

The authors declare that they have no competing interests.

## Authors' contributions

BG carried out all cloning, Tn7 mutagenesis and all sequence analysis, and wrote the paper. CF and CB carried out the Mu and Tn5 mutagenesis and sequencing. NC and BC conceived of the idea and supervised the project. All authors read and approved the final manuscript.
